# An automatic delineation method for bone marrow absorbed dose estimation in ^89^Zr PET/CT studies

**DOI:** 10.1186/s40658-016-0149-0

**Published:** 2016-07-22

**Authors:** N.E. Makris, R. Boellaard, C.W. Menke, A.A. Lammertsma, M.C. Huisman

**Affiliations:** Department of Radiology & Nuclear Medicine, VU University Medical Center, Amsterdam, the Netherlands; Department of Medical Oncology, VU University Medical Center, Amsterdam, the Netherlands; Present address: CREATIS, CNRS UMR 5220, INSERM U1206, F-69677 Bron, France

**Keywords:** ^89^Zr, PET, Active contour, Bone marrow, Absorbed dose

## Abstract

**Background:**

The study aims to develop and validate an automatic delineation method for estimating red bone marrow (RM) activity concentration and absorbed dose in ^89^Zr positron emission tomography/computed tomography (PET/CT) studies. Five patients with advanced colorectal cancer received 37.1 ± 0.9 MBq [^89^Zr] cetuximab within 2 h after administration of a therapeutic dose of 500 mg m^−2^ unlabelled cetuximab. Per patient, five PET/CT scans were acquired on a Gemini TF-64 PET/CT scanner at 1, 24, 48, 96 and 144 h post injection. Low dose CT data were used to manually generate volumes of interest (VOI) in the lumbar vertebrae (LV). In addition, LV VOI were generated automatically using an active contour method in a low dose CT. RM activity was then determined by mapping the low dose CT-derived RM VOI onto the corresponding PET scans. Finally, these activities were used to derive residence times and, subsequently, the self and total RM absorbed doses using OLINDA/EXM 1.1.

**Results:**

High correlations (*r*^2^ > 0.85) between manual and automated VOI methods were obtained for both RM activity concentrations and total absorbed doses. On average, the automatic method provided values that were lower than 5 % compared to the manual method.

**Conclusions:**

An automated and efficient VOI method, based on an active contour approach, was developed, enabling accurate estimates of RM activity concentrations and total absorbed doses.

**Electronic supplementary material:**

The online version of this article (doi:10.1186/s40658-016-0149-0) contains supplementary material, which is available to authorized users.

## Background

In radio-immunotherapy studies, red bone marrow (RM) often is the dose-limiting organ [1]. RM is a complex and dynamic organ, which is distributed in different sites throughout the human body with the axial skeleton (i.e. vertebrae) being the main site. Positron emission tomography/computed tomography (PET/CT) allows for assessment of RM radioactivity concentrations as function of time and thus for estimation of absorbed radiation doses. Conventionally, the plasma time-activity curve is multiplied by a fixed factor (0.19) to estimate the RM time-activity curve [2, 3]. However, there is increasing evidence that the use of a fixed factor may be erroneous, because the RM to plasma activity concentration ratio (RMPR) is not constant and indeed increases over time [4–6]. Consequently, a plasma-based approach could lead to underestimation of RM absorbed dose. Recent findings [7] showed that the use of a standard peripheral blood sampling method may underestimate radiation absorbed dose to RM in patients undergoing [^131^I] rituximab radio-immunotherapy. Therefore, an image-based method should be investigated as an alternative to the blood (or plasma)-based methods.

In order to derive RM activity or dose estimates, a manual RM volume of interest method (VOI_RM_), either defined directly onto the PET image or in a coregistered CT image, is used frequently, as it is simple and straightforward. However, it is also time consuming and labour intensive, especially when analysing several scans per patient. Therefore, an automatic VOI_RM_ method would be advantageous, as it may be both time efficient and observer independent. Such a method could be based on the use of a single Hounsfield unit (HU) threshold value applied to the CT image, allowing for bone marrow activity estimation from the coregistered PET image. However, it is well known that the lumbar vertebrae (LV) consists of different components (compact bone, red and yellow bone marrow, extracellular matrix), displaying a large range of HUs. Consequently, applying a threshold to the CT image may not be optimal, as it would not solely extract the RM component of the LV. This limitation might be overcome by using a more sophisticated active contour method [8, 9] that first identifies the outer bone structure of the LV based on local intensity information in a low dose CT (ldCT) image and subsequently partitions the LV between compact bone and bone marrow.

The purpose of the present study was to develop such an active contour method and to validate it against results obtained from manual positioning of regions of interest.

## Methods

### Imaging protocol

Five patients with advanced colorectal cancer were included. Patients received 37.1 ± 0.9 MBq [^89^Zr] cetuximab within 2 h after administration of a therapeutic dose of 500 mg m^−2^ unlabelled cetuximab. Per patient, five PET/CT scans were acquired on a Gemini TF-64 PET/CT scanner (Philips Healthcare, Cleveland, OH, USA) at 1, 24, 48, 96 and 144 h post injection, respectively. PET data were normalized, corrected for decay, randoms, dead time, scatter and attenuation, and reconstructed using a time-of-flight list-mode ordered-subset expectation maximization reconstruction algorithm using an image matrix size of 144 × 144 and a voxel size of 4 × 4 × 4 mm^3^. In addition, for each time point, a 50-mAs ldCT scan was acquired for attenuation correction purposes. The corresponding ldCT images were reconstructed using an image matrix size of 512 × 512 and a voxel size of 1.17 × 1.17 × 5.00 mm^3^. The study was approved by the Medical Ethics Review Committee of the VU University Medical Center, and informed consent was obtained from each patient prior to inclusion in the study.

### Delineation methods

#### Manual positioning

**Fig. 1:**
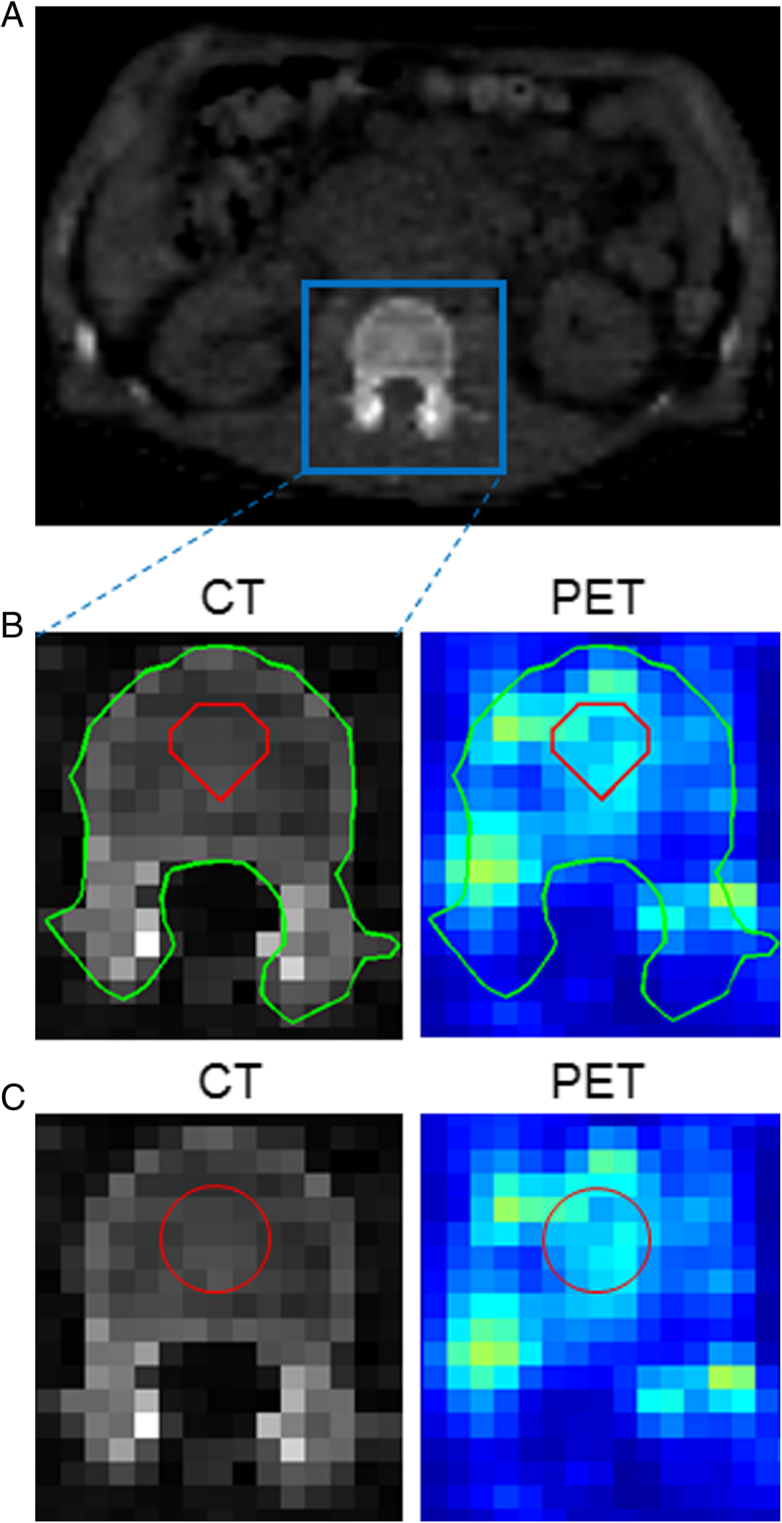
Use of a low dose CT image (**a**) to derive active (**b**) and manual (**c**) contours in one CT slice which are then projected onto the coregistered PET image to determine radioactivity concentration of the enclosed volumes of interest. In each CT slice, a loose region (c_1_—*blue*), enclosing the LV segment, was defined as the starting point of the active contour algorithm. In the next step, the active contour method was applied so as to identify the outer bone contour (c_2_—*green*) of the LV. Lastly, erosion of c_2_ was performed by sampling three pixels (vertically and horizontally) so as to exclude the compact bone from the LV segment, allowing for the creation of a contour that encompasses only the bone marrow component (c_3_—*red*)

Low dose CT scans were first rebinned (with in-house developed software) using trilinear interpolation with a 4 × 4 × 4 mm^3^ voxel size in order to match matrix and voxel size of the PET images (Fig. 1a). Circular regions of interest (1.9-cm diameter) were positioned (in five slices per LV segment) in the bone marrow of all five LV segments by an experienced radiologist, and this was repeated for all five ldCT scans of each patient (Fig. 1c). Regions of interest were positioned in superimposed CT and PET so as to use complementary visual information, and five (cylindrical) VOI_RM_ (diameter 1.9 cm, height 2 cm) of a total volume of 30 mL were extracted per ldCT scan. Subsequently, RM activity was determined by mapping the ldCT-derived manual VOI_RM_ onto the corresponding PET scan.

#### Automatic delineation (active contour)

In a first initialization step, a region with a margin of about 1 cm was drawn around the five LV segments on the rebinned ldCT image (Fig. 1a). Next, this manual VOI was used to produce an LV binary mask that was applied to the ldCT image for extracting a coarse CT region that contained the LV component (CT_1_). The active contour model (see Additional file 1: Supplementary material) used in this study is primarily based on a work by Chan and Vese [8], with additionally incorporating a regularization term in the energy function that enables more robust segmentation of images with weak object boundaries [9]. Recently, the latter methodology was used by Sambuceti et al. [10] on CT data for extracting the whole bone marrow volume. The active contour model was developed in a MATLAB (MathWorks Inc., Natick, MA, USA) environment and was applied to CT_1_, where the LV bone contour (enclosing compact bone and bone marrow) was identified, providing a CT image of the compact bone and bone marrow (CT_2_). This LV bone contour was eroded to exclude compact bone; thus, a three-pixel layer was removed from the outer bone contour, resulting in a CT image containing only the intraosseous volume (CT_3_). The three-pixel layer was based on an educated guess after testing various pixel layers (from one to four pixels) on their impact in the final bone marrow volume and overlap with manually based ROI. Outer LV bone and intraosseous contours can be seen in Fig. 1b. The automatic delineation per subject (five CT scans) required 25 min on a 32-bit desktop PC using an Intel Core 2 Duo 2.8-GHz CPU with 3.2 GB of RAM, making it three times faster than the manual positioning of ROIs. Additionally, the automatic delineation allows the radiologist to perform in the meantime other clinical tasks.

### Evaluation measures

The performance of each erosion kernel size (large, medium and small) was assessed by means of Dice similarity coefficient (DSC). This metric computes the volume overlap between the manual and automatic VOI as

$$ \mathrm{D}\mathrm{S}\mathrm{C}=\frac{2\left|A\cap M\right|}{\left|A\right|+\left|M\right|} $$

where *A* and *M* correspond to the automatic and manual VOI, respectively.

### Organ dosimetry

After determining the mean activity concentration in a VOI_RM_ at all five time points, RM time-activity curves were generated. Other organs were delineated semi-automatically to derive organ time-activity curves [11]. Cumulated activities were calculated as areas under the curves of RM and organ time-activity data by using the trapezoidal rule and assuming physical decay after the last measurement. The latter is a valid assumption, as in the supplementary material it is shown that the bone marrow activity concentration decreases with an effective half-life (73 h) that is shorter than the physical half-life of ^89^Zr (78.41 h). The residence time in the remainder of the body was calculated as the maximum possible residence time assuming physical decay only (no biological clearance) minus the sum of residence times of source organs. The organ residence times were scaled according to patient-specific weight data. Dose conversion factors (*S* values) were taken from OLINDA/EXM 1.1 software and were used for calculation of organ and RM absorbed doses [12]. Red bone marrow residence time data based on the plasma method were taken from Makris et al. [5], and the *S*_RM←RM_, *S*_RM←RB_ and reference man/woman RM volume values used in the calculation of (self and total) RM absorbed dose were taken from OLINDA/EXM 1.1. *S*_RM←RM_ corresponds to the ‘red marrow to red marrow’ contribution, and *S*_RM←RB_ corresponds to the ‘remainder of the body to red marrow’ contribution. As this study focused on RM, only self and total (including contributions from source organs) RM doses will be reported.

## Results

**Fig. 2:**
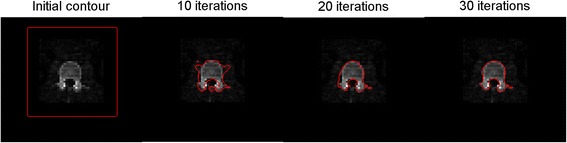
Example of the active contour evolution process from initial to final contour, depicting the detection of the outer bone contour of lumbar vertebrae in a single CT slice

**Fig. 3:**
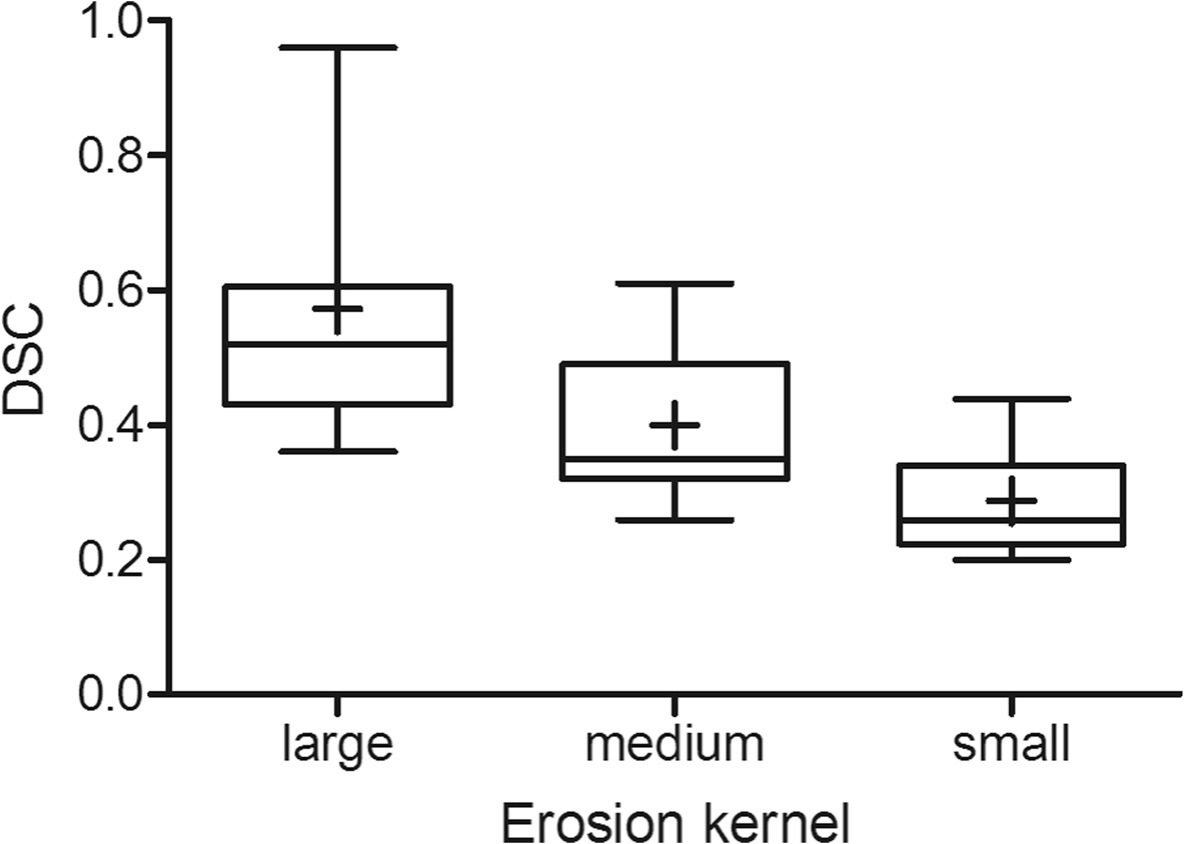
Box plots showing the DSC for three erosion kernel sizes (large: three-pixel; medium: two-pixel; small: one-pixel). The mean is illustrated by a *cross*, median by the *midline*, first and third quartiles by the *lower* and *upper lines* of the box, and min/max by *whiskers*

**Fig. 4:**
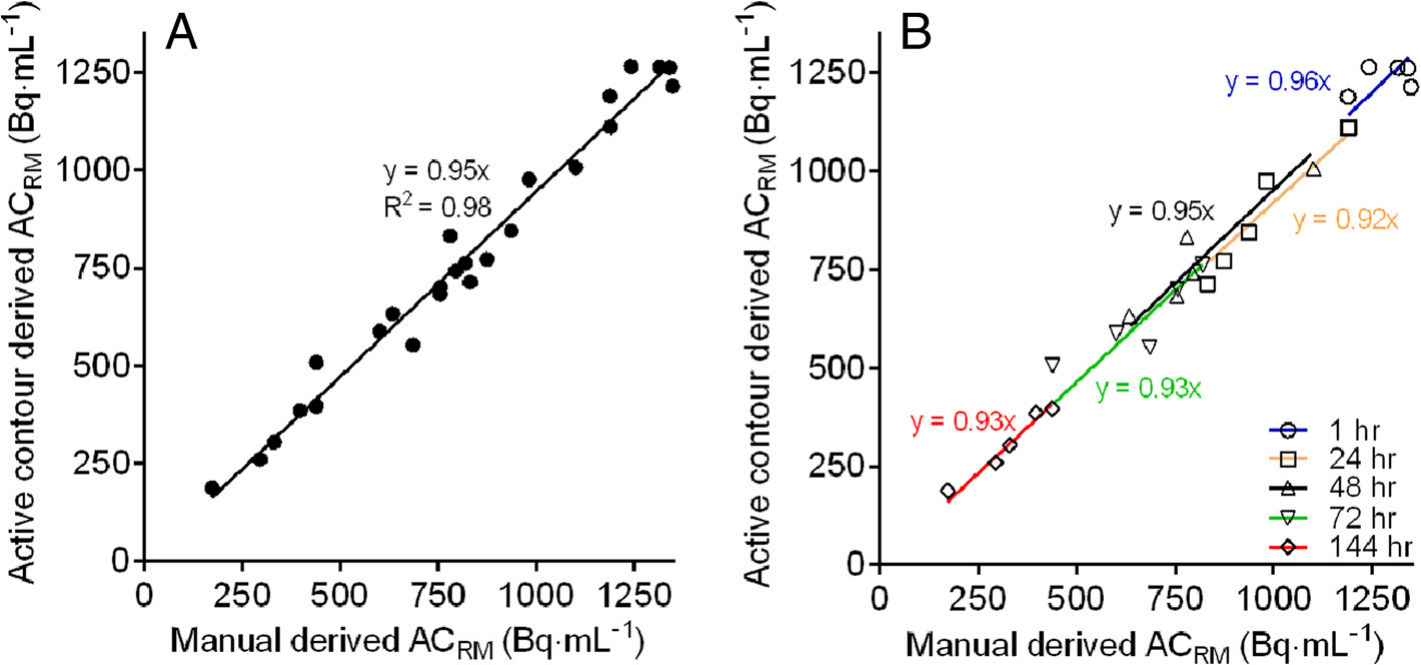
Correlation between manually and automatically (large erosion kernel) derived red bone marrow activity concentrations (Bq × mL^−1^) for all patients (**a**) and per time point across all patients (**b**). The intercept was set to (0,0)

Figure 2 shows a typical example of LV contour detection using an iterative optimization scheme in a CT slice. The number of iterations was 30, as proposed by Li et al. [13]. Figure 3 shows the average DSC obtained using three erosion kernel sizes. DSC values deviated significantly between the large erosion kernel (0.57 ± 0.18; *p* < 0.001) and the other two (medium; 0.40 ± 0.11, small; 0.29 ± 0.08). High correlations between manually and automatically derived RM activity concentration estimates were obtained, as shown in Fig. 4a for all patients and time points and in Fig. 4b per time point across all patients. The automatic method showed a slight underestimation (5 %) in RM activity concentrations compared with the manual method.

**Fig. 5:**
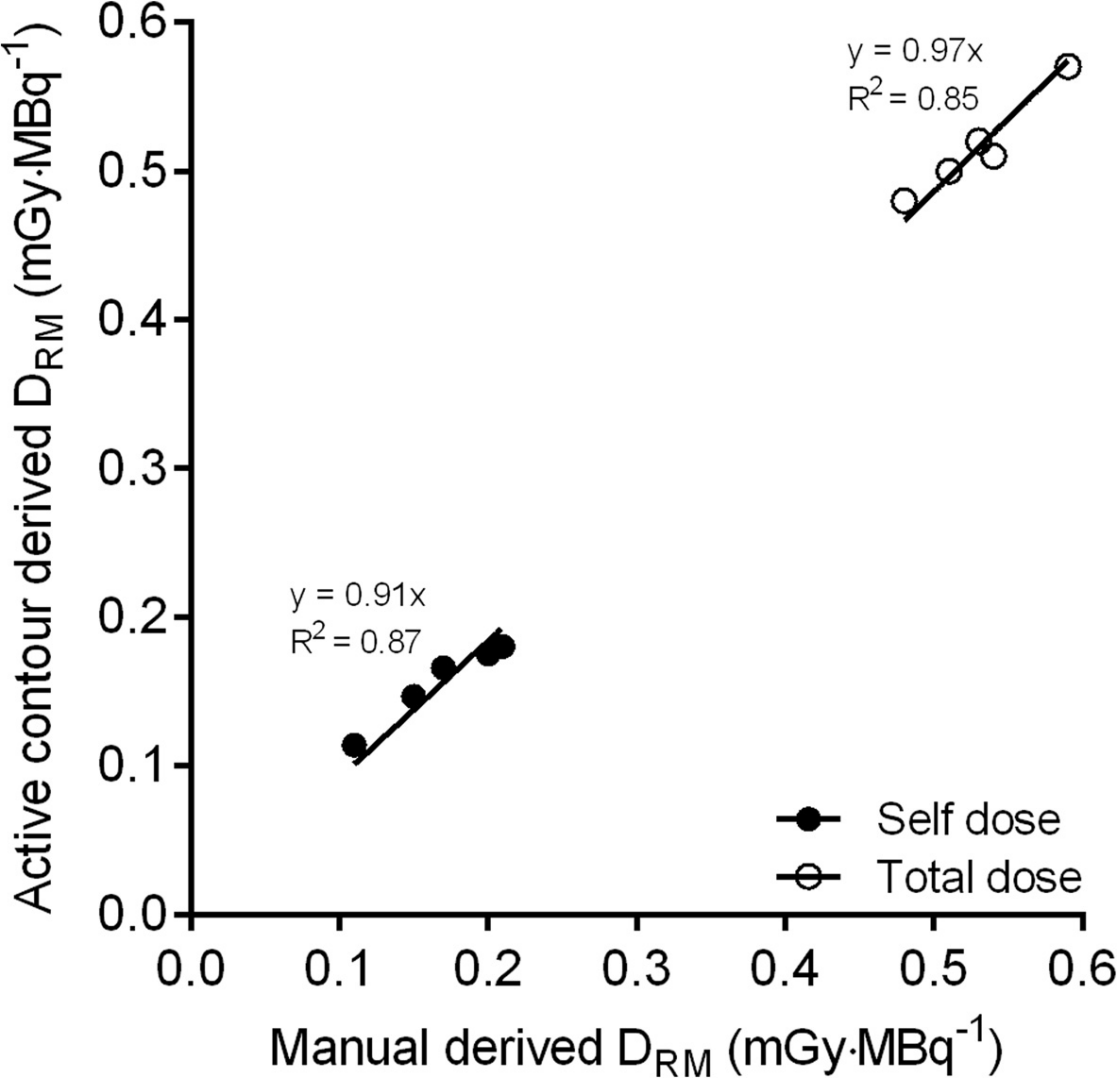
Red bone marrow dose estimates based on manual positioning of ROIs and automatic delineation for self (*closed circles*) and total dose (*open circles*)

For both self and total RM absorbed doses, correlations between the two methods were higher than 0.85 (see Fig. 5). Use of the automatic method resulted in self and total absorbed doses that were on average 8 and 3 % lower, respectively, as compared with the manual method. Table 1 summarizes RM absorbed (self and total) dose estimates for manual, automatic and plasma-based approaches. Significant differences (*p* < 0.05) were seen between both image-based approaches and the plasma-based method in estimating self RM absorbed dose (Table 1).

**Table 1:** RM absorbed dose

	Self RM dose (mGy MBq^−1^)	Total RM dose (mGy MBq^−1^)
Manual positioning	0.17 ± 0.04	0.51 ± 0.04
Active contour	0.16 ± 0.03	0.49 ± 0.03
Plasma method	0.11 ± 0.03	0.45 ± 0.04

## Discussion

Automatically derived RM activity concentrations were within 5 % of manually derived estimates, indicating that the active contour method can identify the intraosseous activity concentration in the LV sufficiently accurately. As a result, accurate estimation of RM absorbed doses could be achieved, showing only a small discrepancy of about 3 % in total dose compared with the manual method. In immunotherapy, where pure or nearly pure *β* emitters are used, only the self RM absorbed dose component contributes to the total RM dose whereas in immunoPET both the self (electrons) and cross (photons) RM absorbed dose contribute to the total RM absorbed dose.

Bone marrow biopsies have been used as the gold standard for assessing radioactivity concentration in the RM. Other procedures, however, that allow assessment of RM activity concentration based on the blood sampling or the PET image have been preferred due to their minimal or non-invasive nature. Yet, several studies reported that a plasma-based approach may not be reliable for accurately estimating RM absorbed doses, as it assumes a fixed RMPR over time [4–6]. To be more specific, in our previous work, it was found that a plasma-based approach can underestimate RM absorbed dose by 40 % (immunoPET setup) and 20 % (radio-immunotherapy setup) when compared with an image-based method [5]. In addition, the use of manual-based VOI_RM_ showed that RMPR increases as function of time. Consequently, image-based methods for estimation of RM absorbed doses may be preferred. However, manual positioning of ROI_RM_ is time consuming, making the development of an automated tool highly desirable. The present study showed that automatic VOI_RM_, when using a large erosion kernel, overlapped approximately 60 %, on average, with the manual VOI_RM_. Additionally, automatic VOI_RM_-based RM doses were comparable to those obtained from manual VOI_RM_ and thus allows for reliable image-based RM dose estimates. As expected, plasma-based RM absorbed doses deviated significantly from both image-based RM absorbed doses.

There are, however, some practical issues and limitations regarding the use of the present automated method. First, there is a pre-processing step in which the user needs to (roughly) extract the LV-associated part of the CT image. Secondly, in a small proportion (15 %) of the automatically generated VOI_RM_, minimal manual adjustments were needed in order to remove voxels (from the lower part of LV segments) that did not represent RM volume. Finally, it was assumed that ^89^Zr activity was distributed homogeneously throughout the intraosseous volume (red and yellow marrow) of the LV. However, yellow marrow (adipose tissue), a non-hematopoietically active tissue, is considered to be a marrow component with reduced activity concentration compared to RM. Additionally, the distribution of the different BM types is dependent upon the skeletal part analysed, RM in the LV is substantially higher than in the shafts of the long bones, i.e. 90 vs 10 % of the intraosseous volume, respectively [10, 14], whereas bones in the appendicular part of the skeleton is mainly occupied by yellow marrow. Consequently, the assumption of homogeneous distributed ^89^Zr activity in the intraosseous volume of the LV would minimally affect the RM activity concentration estimation, thus, with no notable implications for the conclusions of this study.

## Conclusions

A time-efficient and observer-independent method, for estimation of RM activity concentration in the lumbar vertebrae, was developed. The method is based on an active contour approach, providing accurate estimates of RM total absorbed doses.

## Additional file

Additional file 1: Supplementary material. (DOCX 71 kb)
