# Supplementary material for: An automatic delineation method for bone marrow absorbed dose estimation in 89Zr PET/CT studies
Source: EJNMMI Phys. 2016 Jul 22;3:13. doi: 10.1186/s40658-016-0149-0 (PMC4958083; doi:10.1186/s40658-016-0149-0)
Supplement: Supplementary file 1 — Supplementary material. (DOCX 71 kb) [file 40658_2016_149_MOESM1_ESM.docx]

**Supplementary material**

An energy functional is defined in terms of a contour and two fitting functions that approximate the image intensities inside and outside the contour. The energy is then used into a variational level set formulation (see equation 7) with a arc length term (a) which is used for maintaining the regularity of the contour and a level set regularization term (b) that serves in maintaining the regularity of the level set function. Subsequently, a curve evolution equation is derived so as to minimize the associated energy functional. Intensity information in local regions at a certain scale is used to compute the two fitting functions (__ and__) and thereby progressively adapt the contour toward the LV bone boundaries. The energy criterion is as follows:

$\begin{matrix} E(\varphi)=\lambda_{1}\int_{\Omega} e_{1}(x)H(\varphi(x))dx+\lambda_{2}\int_{\Omega} e_{1}(x)(1-H(\varphi(x))) \\ +\nu\int_{\Omega} \delta(\varphi(x))\left\|\nabla\varphi(x)\right\|dx+\mu\int_{\Omega} \frac{1}{2}(\left\|\nabla\varphi(x)\right\|-1)^{2}dx \end{matrix}$ (1)

where λ_1_ > 0, λ_2_ > 0, ν > 0, μ > 0 are fixed parameters, $\varphi$ is the level set function, $\delta$ is the smoothed Dirac function given by the following formula:

$\delta_{\varepsilon}(x)=\frac{\varepsilon}{\pi(x^{2}+\varepsilon^{2})}$, $\varepsilon=1$ (2)

and __ and __ are given by the following formulas:

$e_{i}\left(x\right)=\int K_{\sigma}(x-y)\left|I\left(x\right)-f_{i}\left(y\right)\right|^{2}dy,$ $i=1,2$ (3)

$K_{\sigma}$is a Gaussian kernel defined as:

$K_{\sigma}(u)=\frac{1}{(2\pi)^{n/2}\sigma^{n}}e^{\left\|u\right\|^{2}/2\sigma^{2}}$ (4)

$f_{1}(x)$ and $f_{2}(x)$ approximate image intensities outside and inside the active contour and are given by the following equations:

$f_{1}\left(x\right)=\frac{H_{1}\left(\varphi\left(x\right)\right)I\left(x\right)}{H_{1}\left(\varphi\left(x\right)\right)}$, $f_{2}\left(x\right)=\frac{\left(1-H_{2}\left(\varphi\left(x\right)\right)\right)I\left(x\right)}{\left(1-H_{2}\left(\varphi\left(x\right)\right)\right)}$ (5)

$I(x)$ are the intensities used for the fitting energy, and $H(x)$ is a regularised version of the Heaviside function:

$H(x)=\frac{1}{2}\left[1+\frac{2}{\pi}\arctan\left(\frac{x}{\varepsilon}\right)\right]$ (6)

Minimization of the energy functional can be obtained by solving the evolution problem:

$\frac{\partial\varphi}{\partial t}=-\delta\left(\varphi\right)\left(e_{1}-e_{2}\right)+\delta\left(\varphi\right)div\left(\frac{\nabla\varphi}{\left|\nabla\varphi\right|}\right)+\left(\nabla^{2}\varphi-div\left(\frac{\nabla\varphi}{\left|\nabla\varphi\right|}\right)\right)$ (7)

(a) (b)


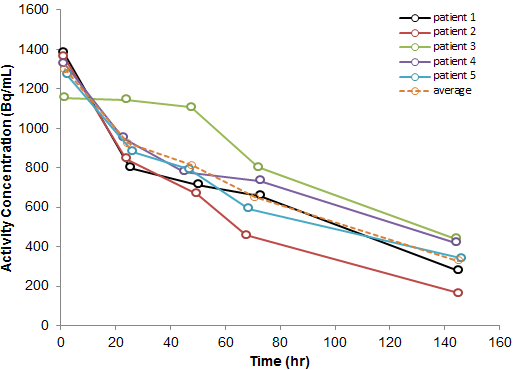


Supplemental figure: Non-decay corrected bone marrow time-activity curves (TAC). The dashed line shows the average TAC with values of 1300 Bq/mL at 1hr to 330 Bq/mL at 145hr, post-injection. This can be translated to a half-life of 73 hr which means that the compound decays slightly faster than its physical decay (78.41 hr).
